# First person – Kazuki Omata

**DOI:** 10.1242/bio.060075

**Published:** 2023-08-09

## Abstract

First Person is a series of interviews with the first authors of a selection of papers published in Biology Open, helping researchers promote themselves alongside their papers. Kazuki Omata is first author on ‘ Isolation and evaluation of erythroid progenitors in the livers of larval, froglet, and adult *Xenopus tropicalis*’, published in BiO. Kazuki is a PhD student in the lab of Takashi Kato at Waseda University, Japan, investigating the difference in the function and production mechanisms between mammalian enucleated erythrocytes and non-mammalian nucleated erythrocytes.


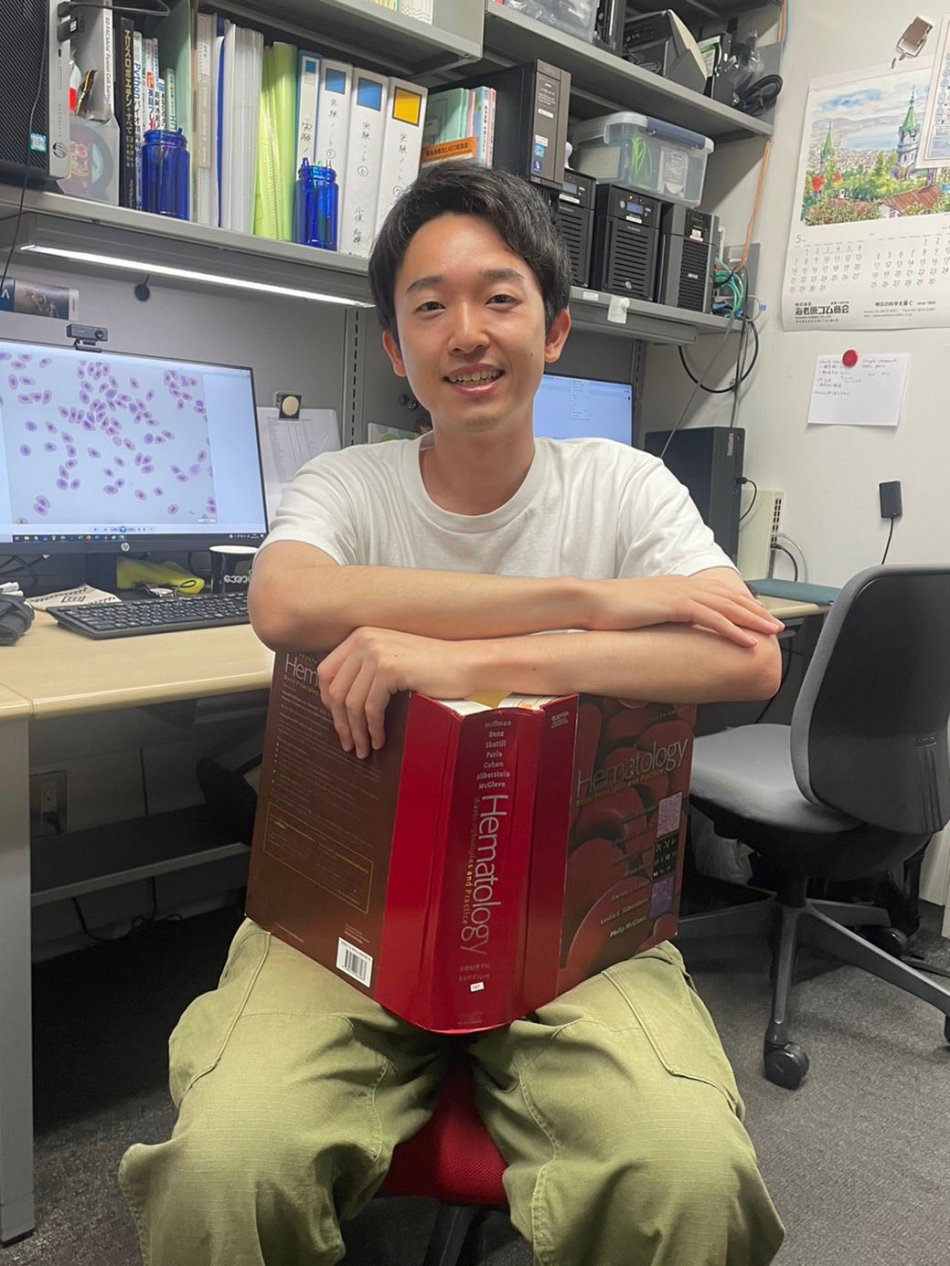


**Kazuki Omata**

**Describe your scientific journey and your current research focus**

After learning that one million red blood cells are produced per second, I developed an interest in hematology. Through laboratory training in the current laboratory of Professor Takashi Kato, I became even more fascinated by the universality and the diversity of hematopoiesis conserved among vertebrates. The focus of the present work is the progenitor of red blood cells. In the next study, I will investigate the signaling pathway in the red blood cell.

**Who or what inspired you to become a scientist?**

I was inspired by Jean-Henri Casimir Fabre's ‘Souvenirs entomologiques’ and developed a fascination for biology. Currently, my research is focused on vertebrates, but I still love to learn about the way all living things live.

**How would you explain the main finding of your paper?**

In short, we have established a tool and a method to count the cells that become red blood cells in frogs. There are many differences between humans and frogs, and one of the most important differences is metamorphosis. Frogs undergo dynamic changes from larvae to adults, whereas humans do not. Despite these differences, the systems necessary for survival are similar between frogs and humans, including the blood system. Within the unique biological phenomenon of frog metamorphosis, the mechanism of red blood cell production, which is shared with humans, is not fully understood. Therefore, we developed an antibody that can be used to count frog red blood cells. In this paper, we demonstrated the changes in the number of cells that become red blood cells in frogs during metamorphosis. This finding will highlight the difference in the blood production system between humans and frogs.

**What are the potential implications of this finding for your field of research?**

This work has provided insights into how three different axes – individuals, organs, and functions – are controlled through metamorphosis and subsequent maturation. Interestingly, humans undergo hematopoiesis in the liver during the fetal period but lose this ability immediately after birth and the bone marrow becomes the major site of hematopoiesis. The method reported in this paper is expected to provide a means to investigate the mystery of why hematopoietic capacity in the liver is lost in humans while maintained in frogs. In addition, the established antibody against the frog molecule can inhibit signaling molecules critical for erythropoiesis. By using this antibody, there is an opportunity to discover unique biological phenomena that have not been found in mice or humans.

**Figure:**
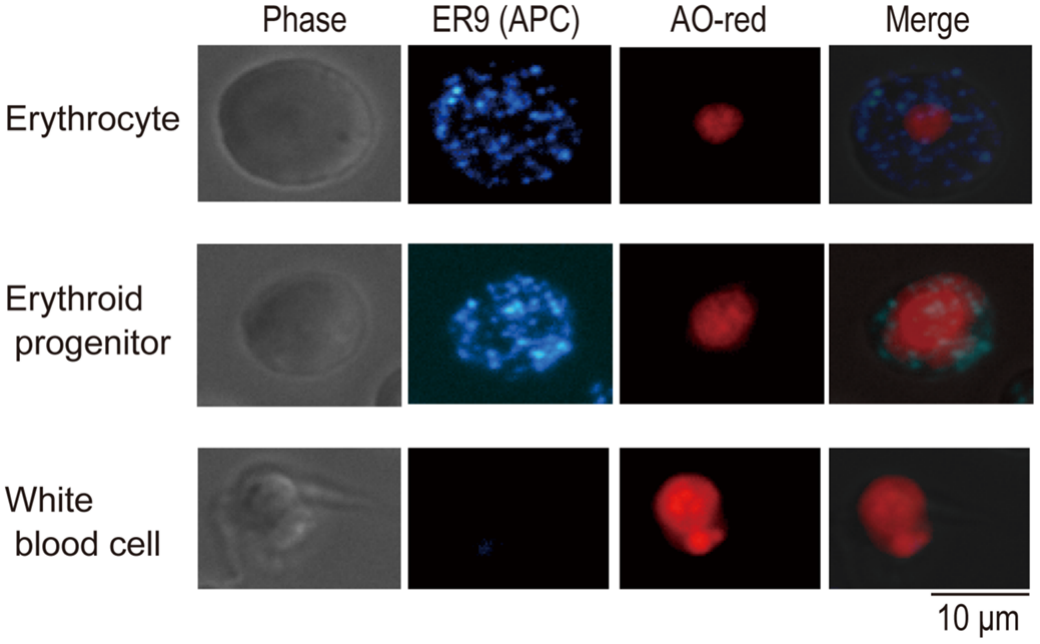
*Xenopus* erythrocytes immunostained by the monoclonal antibody against erythropoietin receptor.

**Which part of this research project was the most rewarding?**

One of the important parts is antibody generation. To be honest, this part was mainly done by a co-author who worked in our lab about ten years ago. I was not there when the activity of the antibody was first recognised. In writing the paper using this antibody, I had the opportunity to re-examine the data and refer to my senior colleagues' experimental notes and previous presentations, which reminded me of the importance of experimental records.

**What do you enjoy most about being an early-career researcher?**

I think that early-career researchers are able to concentrate and work on a single project. By having the time to think deeply about a question, I feel lucky to be able of the imagine the pure joy of biology.

**What piece of advice would you give to the next generation of researchers?**

In the future, there will probably be more opportunities for researchers to explain or present something to people without a scientific background. Therefore, I think it is important to proactively learn how to speak in a clear and organised manner, how to create videos, and how to use social media effectively, even if it is only on a small scale.

…I think it is important to proactively learn how to speak in a clear and organised manner, how to create videos, and how to use social media effectively…

**What's next for you?**

First and foremost, I will complete my doctoral thesis. After obtaining PhD, I aspire to challenge myself by collaborating with individuals who have an engineering background in a company where I can utilise my background in biology to engage in product development.
